# Supplementary material for: MetaRibo-Seq measures translation in microbiomes
Source: Nat Commun. 2020 Jun 29;11:3268. doi: 10.1038/s41467-020-17081-z (PMC7324362; doi:10.1038/s41467-020-17081-z)
Supplement: Supplementary file 10 — Supplementary Data 7 [file 41467_2020_17081_MOESM10_ESM.zip › File2/Confidence_High_Taxonomy/294536_out.krona.html]

Javascript must be enabled to view this page.

members
magnitude
magnitudeUnassigned
count
unassigned
taxon
rank

294536\_out

2

2
2
superkingdom

phylum
2
976

200643
2
class

order
2
171549

171552
1
family

genus
1
838

species
1262930

SRS016541\_contig\_number\_contig-100\_163.73700
1

1
815
family

genus
1
816

species
1

SRS023914\_contig\_number\_24622
821
